# Effect of Carbon Content on Microstructure, Properties and Texture of Ultra-Thin Hot Rolled Strip Produced by Endless Roll Technology

**DOI:** 10.3390/ma14206174

**Published:** 2021-10-18

**Authors:** Peng Tian, Guoming Zhu, Yonglin Kang

**Affiliations:** School of Material Science and Engineering, University of Science and Technology Beijing, Beijing 100083, China; tpchbr@163.com

**Keywords:** carbon content, texture, ultra-thin, endless roll technology, formability

## Abstract

In order to make a comprehensive comparison between ultra-thin hot rolled low carbon steel (LC) and extra low carbon steel (ELC) produced by endless roll technology and explain the differences, a detailed investigation into the microstructural characterization, characteristics of cementite and precipitates, mechanical properties, internal friction peaks, texture characterization by an X-ray powder diffractometer and electron backscatter diffraction, and formability including earing behavior, hole expanding ratio and V-shaped bending properties was carried out with different carbon content for 1.0 mm thickness ultra-thin hot rolled strip produced in endless strip production line. The experimental results indicate that the microstructure of both is composed of multi-layer areas with different grain sizes and thicknesses, the strength and elongation of LC are higher than that of ELC, but the content of solid solution carbon atoms and r of ELC are higher than that of LC, at the same time, the formability of ultra-thin strip ELC is better than that of LC mainly related to the content of {hkl} <110> and {111} <112> of ELC was higher than those of LC. The mechanical and formability properties of ultra-thin hot rolled strip by endless roll technology can meet the requirements of replacement cold rolled strip by hot rolled strip.

## 1. Introduction

The market of flat steels is changing on the one hand to lower average thicknesses and advanced steel design [1] and on the other hand it needs more high-quality and low-cost green products. The most radical way and highest potential is given by directly delivering hot rolled products as cold rolled substitutes or high-strength thin-gauge products as conventional thick-gauge substitutes. Endless rolling technology is a new short process endless rolling thin and wide strip steel production technology in recent years. It plays a leading and exemplary role in low cost, energy conservation and emission reduction and green manufacturing because it has the characteristics of short process, near net shape and endless rolling production. Thus, endless strip production (ESP) is the most advanced endless rolling technology in the world today and has been honored as the third technological revolution in steel industry [2] marked a new era of mass production of thin gauge (thickness ≤ 2.0 mm) and ultra-thin gauge (thickness ≤ 1.5 mm) hot rolled production. The ESP process is characterized in that the thin slab after continuous casting directly enters rough rolling without heating, and an inductive equalization furnace is added between finish rolling and rough rolling to realize rapid temperature rise of the intermediate slab by 200–300 °C. Endless rolling technology is conducive to energy conservation and emission reduction and carbon neutralization as soon as possible.

At present, there is little research on ultra-thin gauge hot rolled plate [3], because it is difficult for conventional hot rolling line to produce ultra-thin hot rolled strip less than 1.5 mm or 1.2 mm [4]. Although ESP can produce 0.6 mm super-thin gauge (thickness < 1.0 mm) hot rolled strip, and 1.0 mm ultra-thin gauge hot rolled plate has realized mass production and application [5]. In addition, the research was mainly focused on the mechanical properties of more than 1.2 mm thickness hot rolled strip [6] and the extremely-thin gauge (thickness < 0.5 mm) cold rolled and annealed plate [7] with little attention on the texture and formability of the ultra-thin hot rolled plate. The physical metallurgical process and thermal history of hot wide strip steel produced by different technological processes are obviously different even with the same chemical composition, the technological process difference will affect the microstructure transformation, grain size, morphology and distribution of precipitated particle, so as to affect the final performance of the strip [8]. In this paper, the extra low carbon steel (ELC, C = 0.008–0.025%) and the low carbon steel (LC, C = 0.026–0.06%) were produced by ESP line, the microstructure, mechanical properties, texture and formability of the two ultra-thin hot rolled steel plate are comparative investigated. This study will deepen the understanding of ultra-thin hot rolled plate, promote the technology development and product application of replacement of cold rolled strip by hot rolled strip.

## 2. Materials and Methods

For this study, two ultra-thin hot rolled aluminum-killed steel strips with 1.0 mm thickness manufactured by ESP line were obtained. The chemical composition of two steels were listed in Table 1, differing in C content.

**Figure 1:**
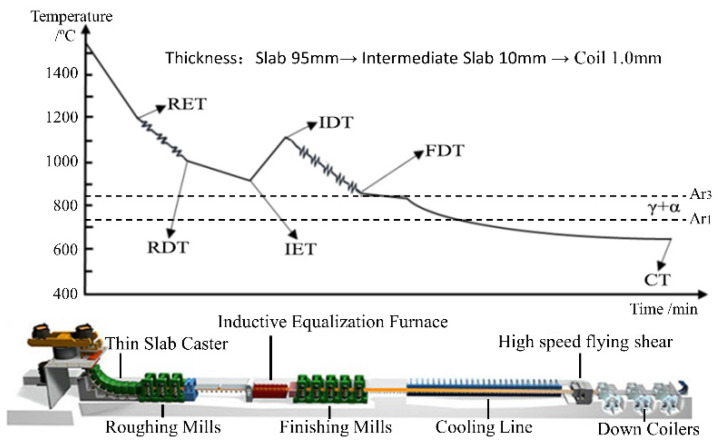
Layout and process diagram of the ESP line.

In ESP line, the thin continuous casting slab with 95 mm thickness after thin slab caster directly enters the roughing mills of three-stand high reduction mills and is rolled into intermediate slab with 10 mm thickness, which is then heated to about 1110 °C by inductive equalization furnace with a short distance of less than 12 m, and then rolled by five-stand finishing mills, after cooing line, high speed flying shear and down coiler, the coil with 1.0 mm thickness is obtained. The layout [5] and process diagram of ESP line is shown in Figure 1, in which, roughing mill entry temperature, roughing mill delivery temperature, inductive equalization furnace entry temperature, inductive equalization furnace inductive equalization furnace temperature, finishing mill delivery temperature, and coiling temperature are denoted by RET, RDT, IET, IDT, FDT, and CT, respectively.

The process parameters of the ESP line for 1.0 mm thickness are shown in Table 2, the process parameters for ELC and LC are considered to be the similar.

Specimens were cut from quarter part of the transversal of coils. According to the standard of GB/T 228.1–2010, the width of P2 standard tensile specimen is 12.5 mm and the original gauge length is 50 mm, the tensile tests at room temperature were performed on the tensile testing machine (MTS810, Minneapolis, MN, USA) at a strain rate of 3 × 10^−3^ s^−1^. Samples surfaces along the thickness direction were mounted, polished, and then etched in 4 vol% nitric solution to observe the microstructure under the optical microscope (OM) (Nikon eclipse LV150, Nikon corporation, Tokyo, Japan) and the scanning electron microscopy (SEM) (Quanta FEG450, FEI corporation, Hillsboro, OR, USA). The dislocation and the carbon extract coated samples were examined by the transmission electron microscope (TEM) (Hitachi H-8100, Hitachi, Tokyo, Japan). The internal friction of the two steels were measured by Multi-Function Internal Friction Appa Ratus (MFP-1000, Chinese Academy of Sciences, Hefei, China), the samples were cut into cuboids with dimensions of 1 mm (thickness direction) × 3 mm (width direction) × 50 mm (rolling direction), and all surface were polished. During the internal friction experiment, the heating rate is 2 °C/min, the vacuum degree is less than 4 × 10^−3^ Pa, the test temperature is 0–120 °C, and the measurement method adopts free attenuation mode. The macroscopic texture distribution was determined by x-ray powder diffractometer (XRD) (MXP21VAHF, Mac, Tokyo, Japan). The preparation for electron backscatter diffraction (EBSD) samples includes normal mechanical polishing and the electrolytic polishing with the solution containing 5 vol. % perchloric acid and 95 vol.% acetic acid for 10–25 s. Earing tests were carried out on a universal sheet-testing machine (BCS-30, Beihang, Beijing, China). The preparation of samples, operation of earing test and treatment of test results are in accordance with the national standard of GB/T 15825.7-2008. Hole expanding test were carried out on a punching machine (TGCK-600, Tiger Running, Beijing, China) and a reaming testing machine (TGUP-300, Tiger Running, Beijing, China) accordance to GB/T 15825.4-2008. The bending test was carried out under the self-made 60° V-shaped device [9] and the above tensile testing machine. The precipitates were determined by small angle X-ray scattering (SAXS) (X’Pert PRO Nano-1 2B, Panalytical, Almelo, Netherlands), select the Cu Ka radiation, and set the generator to 40 kV and 40 mA. The average grain size was measured and calculated according to GB/T 6394-2017, the thickness was based on the scribed line and the substrate surface, and the precipitate was based on its boundary, the particle size distribution calculation software of Nano Measure 1.2 was used to measure the thickness and precipitate or grain size.

## 3. Results

### 3.1. Microstructural Characterization

**Figure 2:**
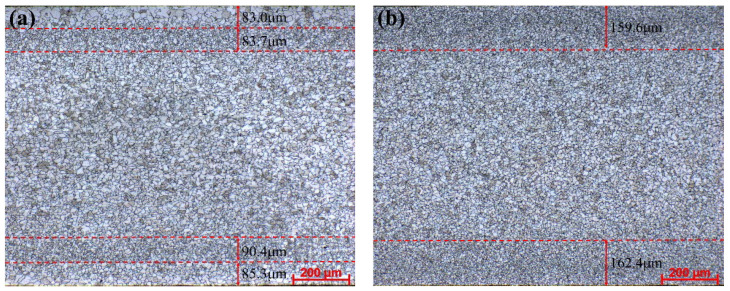
OM microstructure of different C content for 1.0 mm thickness. (**a**) ELC; (**b**) LC.

Figure 2 shows the full thickness microstructure of the transverse section. By comparing the microstructure of different C content, it can be seen that there are significant differences in the distribution of microstructure along the thickness direction and the grain size of ELC is slightly smaller than that of LC. The microstructure in the thickness direction of strip is non-uniform, the grain size distribution of ELC has five-layer structure, while that of LC has only three-layer structure. In Figure 2a the microstructure of ELC from the upper surface to the lower surface along the thickness direction includes an upper surface coarse grain area with a thickness of about 83.0 µm and an average grain size of 20.0 µm, a near upper surface fine grain area with a thickness of about 83.7 µm and an average grain size of 7.4 µm, a central medium grain area with a grain size of about 10.8 µm, a near lower surface fine grain area with a thickness of about 90.4 µm and an average grain size of 7.6 µm, and an lower surface coarse grain area with the thickness is about 85.5 µm and the average grain size is 17.5 µm. In Figure 2b the microstructure of LC includes an upper surface fine grain area with a thickness of about 159.6 µm and an average grain size of 7.1 µm, a central medium grain area with a grain size of about 10.3 µm, and an upper surface fine grain area with the thickness is about 162.4 µm and the average grain size is 6.8 µm.

**Figure 3:**
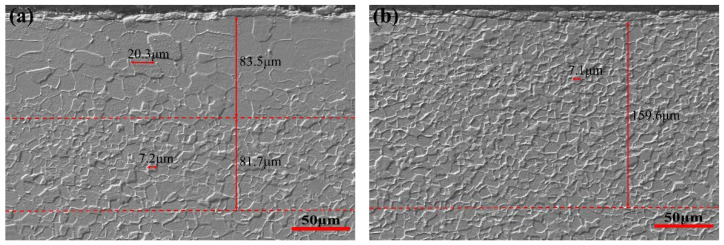

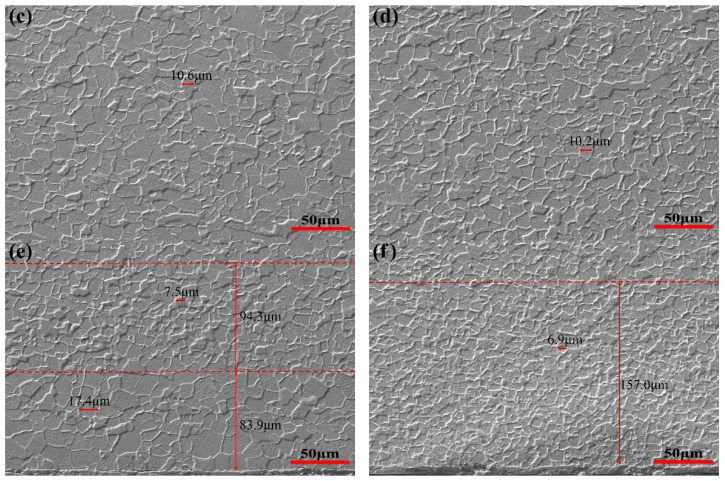
SEM microstructure of different C content for 1.0 mm thickness. (**a**) The upper surface of ELC; (**b**) the upper surface of LC; (**c**) the central thickness of ELC; (**d**) the central thickness of LC; (**e**) the lower surface of ELC; (**f**) the lower surface of LC.

In order to further observe the difference of microstructure between ELC and LC, the SEM microstructures exhibited in Figure 3, consist of the ferrite and a few cementite distributed at ferrite grain boundaries or corners. In Figure 3a the microstructure of ELC includes an upper surface coarse grain area with a thickness of about 83.5 µm and an average grain size of 20.3 µm, and a near upper surface fine grain area with a thickness of about 81.7 µm and an average grain size of 7.2 µm. In Figure 3b the microstructure of LC includes an upper surface fine grain area with a thickness of about 159.6 µm and an average grain size of 7.1 µm. In Figure 3c,d the microstructure of ELC and LC is a medium grain area with average grain sizes of 10.6 µm and 10.2 µm, respectively. In Figure 3e the microstructure of ELC includes a lower surface coarse grain area with a thickness of about 83.9 µm and an average grain size of 20.3 µm, and a near upper surface fine grain area with a thickness of about 94.3 µm and an average grain size of 7.5 µm. In Figure 3f, the microstructure of LC includes an upper surface fine grain area with a thickness of about 157.0 µm and an average grain size of 6.9 µm. Compared with Figure 2, it can be seen that there is little difference in grain size and area thickness, which is mainly related to the magnification and the clarity of grain boundary.

### 3.2. Characteristics of Cementite and Precipitates

According to the Fe-Fe_3_C phase diagram, ELC and LC are located on the left and right sides of point P (maximum dissolved carbon content in α-Fe) respectively due to different carbon content, and their microstructure and phase content will be significantly different. When ELC and LC are slowly cooled from 727 °C to room temperature, because less than 0.0008% C can be dissolved in ferrite at room temperature, excess carbon will precipitate along the ferrite grain boundaries in the form of cementite, which is called Fe_3_C_III_. Similarly, carbon content higher than 0.0218% will undergo eutectoid transformation to form the microstructure of ferrite (α-Fe) and pearlite (P), therefore, in theory, α-Fe, P and Fe_3_C_III_ can be observed in LC at room temperature. According to the lever law [10],

(1) $$\omega \left(A\right)=\frac{x_{b}-x_{p}}{x_{b}-x_{a}},$$

Here, ɷ(A) is the relative quantity of component A, x_a_, x_b_ and x_p_ are the fraction of component A, B and alloy P, respectively.

for ELC: ɷ(Fe_3_C_III_) = (0.012 − 0.0008)/(6.69 − 0.0008) = 0.17%.

for LC: ɷ(Fe_3_C_III_) = (0.0218 − 0.0008)/(6.69 − 0.0008) = 0.31%,

      ɷ(P) = (0.044 − 0.0218)/(0.77 − 0.0218) = 2.97%.

**Figure 4:**
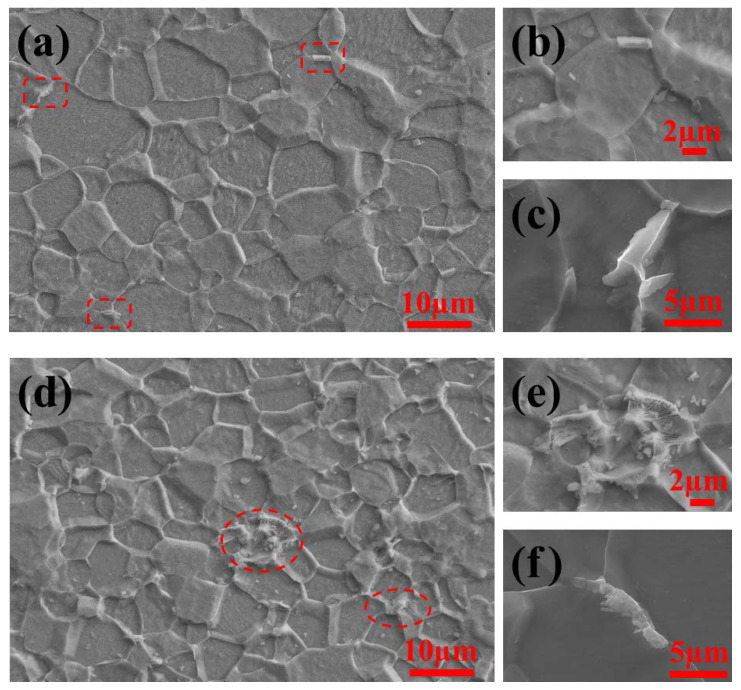
Distribution and morphology of cementite with different C content. ELC: (**a**–**c**); LC: (**d**–**f**).

It can be seen from Figure 4 that there are significant differences in the distribution and morphology of cementite for ELC and LC. In Figure 4a, the microstructure of ELC consists of dispersed ferrite and a small amount of Fe_3_C_III_ (see red rectangulars in Figure 4a). Fe_3_C_III_ in the form of short rod (Figure 4b) and irregularly shape (Figure 4c) are mainly distributed at the boundaries and corners of ferrite. At the same time, there are many nano precipitates in the ferrite (Figure 4c). In Figure 4d, the microstructure of LC consists of dispersed ferrite and a small amount of pearlite (see red ovals in Figure 4d), cementite in cluster pearlite located at the corner of ferrite exists in different pearlite fields in uniform lamellar form (Figure 4e) and cementite in uniform lamellar form is distributed at the ferrite grain boundaries (Figure 4f), similarly, a small number of spherical precipitates exist in the ferrite (Figure 4d,f).

**Figure 5:**
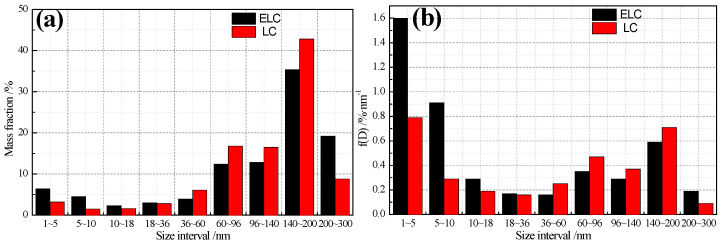
Comparison of particle size distribution with different C content. (**a**) mass fraction; (**b**) mass density.

After the steel strips were corroded by the aqueous solution mixed with 10 g/L ammonium sulfate and 10 g/L citric acid, the particle size of precipitated particles obtained by filtration of the solution was analyzed by SAXS. The mass fraction distribution and mass density distribution under different particle sizes are obtained by SAXS, as shown in Figure 5. The mass fraction of less than 36 nm and >200 nm in ELC is greater than that of in LC, and the mass fraction of 36–200 nm is vice versa. The mass fraction is over 35% in 140–200 nm, the mean size of ELC is 136.3 nm, which is slightly more than 131.4 nm of LC, mainly because the mass fraction above 200 nm in ELC is 118% more than that in LC (Figure 5a). The mass density distribution has the same law as the mass fraction distribution, and the highest mass density is between 1–5 nm.

**Figure 6:**
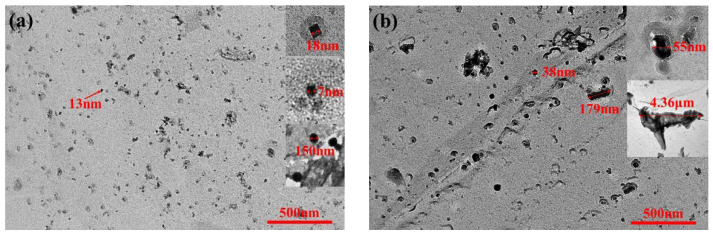
Morphology and distribution of precipitates with different C content. (**a**) ELC; (**b**) LC.

Relevant studies show that nanoscale oxide and sulfide precipitates had been observed in low carbon steel strips produced by CSP, and these small precipitates may contribute to the strengthening and grain refinement under certain conditions [11]. In low-carbon aluminum-killed steel, the second-phase particles frequently discovered are MnS, Al_2_O_3_, Fe_3_C and AlN [12,13]. The morphology and distribution of precipitates observed under TEM after carbon extraction replicas of ultra-thin strip produced by ESP are shown in Figure 6. It can be seen from the comparison that the precipitates of ELC are large in quantity and small in size, mainly square AlN, spherical Fe_3_C and Al_2_O_3_ (Figure 6a), while the precipitates of LC are small in quantity but large in size, mainly large size ellipsoidal MnS and rectangular AlN, in addition, lamellar pearlite with a length of 4.36 µm is observed at the corner of ferrite boundaries (Figure 6b). The result is consistent with Figure 5.

### 3.3. Characteristics of Mechanical Properties

**Figure 7:**
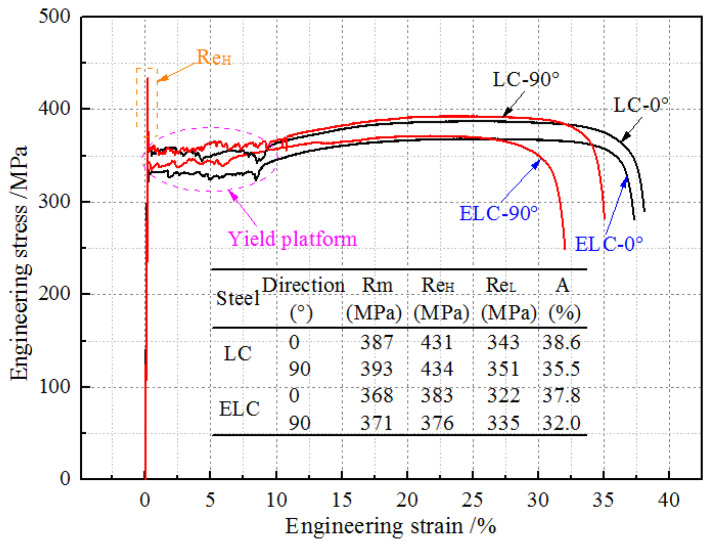
Engineering stress-strain curves and tensile properties of different C content.

The engineering stress-strain curves and tensile properties of the two steels in rolling directions (0°) and transverse directions (90°) are shown in Figure 7. The initial yielding exhibits a material instability known as Lüders banding, which is a dislocation driven phenomenon that macroscopically manifests as inhomogeneous deformation. It exhibits an initial shape stress peak (upper yield stress) followed by a stress plateau (lower yield stress) that extends over a Lüders strain, it is called the discontinuous yield phenomenon. It can be seen that the upper yield strength (Re_H_) (see orange rectangular in Figure 7) of LC is more than 40 MPa higher than the tensile strength (Rm), while that of ELC is only higher than 5 MPa. Rm at 0° is about 5 MPa lower than Rm at 90°, the lower yield strength (Re_L_) at 0° is about 10 MPa lower than Re_L_ at 90°. Each yield platform has obvious zigzag shape and the percentage yield point extension (see purple oval in Figure 7) or Lüders strain is about 10%, but the percentage yield point extension of low carbon steel produced by compact endless casting and rolling process is only 5% [14]. The elongation (A) at 0° is higher than A at 90°, in particular, A at 90° of ELC decreases significantly mainly because the percentage yield point extension reduced by 3%, which needs further study. On the whole, the strength other than Re_H_ of ELC is about 20 MPa lower than that of LC and A of ELC is lower than that of LC. The longer yield platform of ESP steel strip is conducive to improve the elongation of the material and enhance the plasticity, but it also shows that the steel has room temperature aging and is not conducive to stamping forming [15]. Therefore, it is necessary to study the generation mechanism of yield platform and high Re_H_ and their influence on forming properties.

In order to test the stamping performance of steel strip, plastic strain ratio (r), weight average of r values ($\bar{r}$) and degree of planar anisotropy (Δr) are usually used to measure the degree of anisotropy [16]. The r value and $\bar{r}$ are required as high as possible, and Δr as small as possible. Table 3 shows r value and n value for 1.0 mm thickness. It can be seen that r values deviating from rolling direction by 0° (r0), 45° (r45) and 90° (r90), and $\bar{r}$ value of ELC higher than that of LC, but |Δr| of ELC is relatively small, indicating that the ear making is small, that is, the anisotropy of ELC is smaller than that of LC. In addition, the Δr < 0 of the two steels indicates that the ear appears in the direction of 45° to the rolling direction. The n values of the two steels are similar, near 0.22, and the n value of rolling direction is slightly higher than that of transverse direction.

### 3.4. Characteristics of Internal Friction Peaks

**Figure 8:**
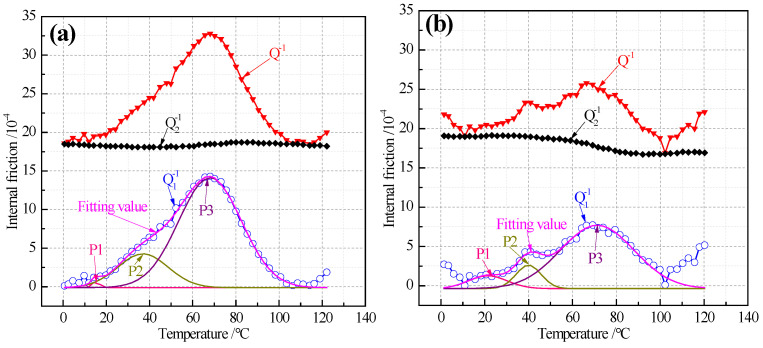
Internal friction spectra of different C content: (**a**) ELC; (**b**) LC.

Because the Snoek relaxation can be used to study the distribution and variation characteristics of C and N atoms in the octahedral gap of ferrite, the internal friction method for measuring Snoek relaxation is a common analytical method for measuring the content of solid solution carbon atoms in iron and steel [17]. The internal frictions—temperature spectrums of ultra-thin hot rolled strip produced by ESP—are shown in Figure 8. $Q^{-1}$ is total internal friction measured by internal friction meter, $Q_{1}^{-1}$ is the real internal friction obtained by calculation, $Q_{2}^{-1}$ is the background internal friction deducted by the calculation software attached to the equipment, and the relationship between them is $Q^{-1}$ = $Q_{1}^{-1}$ + $Q_{2}^{-1}$. Then, the peaks of $Q_{1}^{-1}$ were separated and fitted by OriginPro 8.5. It can be seen from Figure 8 that the real internal friction is composed of three internal friction peaks (P1, P2 and P3) and the peak value increases significantly with the increase of temperature. The peak temperatures of P1, P2 and P3 are about 20 °C, 40 °C and 70 °C, respectively. At the same time, the P2 peak shape and half width of LC are more obvious than those of LC. It can be inferred that there is an internal friction peak above 120 °C because the internal friction increases gradually when the temperature is higher than 100 °C. Relevant studies show that the Snoek peak of solid solution nitrogen atoms appears at about 20 °C [18,19], the Snoek peak of solid solution carbon atoms appears about 40 °C [19,20,21,22,23] or about 67 °C [15,20,24] and the activation energy is about 84 kJ/mol. In SDH3 steel, it is found [25] that the Snoek peak of solid solution carbon atoms is decomposed into two peaks, s1 and s2. The s1 peak is the Snoek peak of solid solution carbon atoms in pure α-Fe, and s2 peak is the Snoek peak of solid solution carbon atoms in body centered cubic lattice in which some Fe atoms are replaced by weak carbide forming elements such as Si and Mn. It is also found [20] that the internal friction spectrum of automotive plates with different stamping properties has obviously different characteristics with different peak numbers. According to these results, it can be preliminarily determined that the peak near 20 °C is the Snoek peak of solid solution nitrogen atoms, and the other two peaks are the Snoek peak of solid solution carbon atoms.

In the binary Fe-C alloy, the height of the Snoek peak ($Q_{\max}^{-1}$) is assumed to be directly proportional to the solid solution atonic content ([C]) [24]. That is,

(2) $$[C]=K\;Q_{\max}^{-1},$$

Here, K is a factor which depends on the grain size, substitutional alloying element and the texture of the alloy. In this experiment, K is recommended to be 1.33 for solid solution carbon atoms and 1.28 for solid solution nitrogen atoms, this value is similar to that in other literatures [26,27]. The solid solution carbon atom content of ELC at P3 is significantly higher than that of LC. The reason is that the long of layer cooling line in ESP is about 40 m and that in the conventional hot rolling line is about 120 m. Therefore, high cooling speed results in supersaturated solution of carbon atoms in ultra-thin strip steel. It is considered that there are two reasons why the content of solid solution carbon atoms in ELC is higher than that in LC. One is that LC undergoes eutectoid transformation to form pearlite, the carbon atoms dissolved in or near the pearlite field are easy to diffuse to the lamellar cementite and precipitate in the form of Fe_3_C_III_ because of the short distance. The other is that the ferrite grains of LC are relatively small, and the content of dissolved carbon in ferrite is nearly twice that of ELC, so these solid solution carbons are also easier to diffuse to the ferrite grain boundary and precipitate in the form of Fe_3_C_III_ or take heterogeneous points as the nucleation core to precipitate spherical cementite in ferrite grains, as shown in Figure 4. The activation energy (H) can be calculated [28] by:

H = RTm In(kTm/hfm) + TmΔs,(3)

where R is the ideal gas constant and R = 8.314472 J/(mol·K), Tm and fm correspond to the internal friction and frequency of the peak, k is Boltzmann constant and k = 1.3806505 × 10^−23^ J/K, h is the Planck constant and h = 6.62607015 × 10^−34^ J·s, Δs is the activation entropy and Δs = 1.1 × 10^−4^ eV/K. Table 4 shows the important indexes characterizing the internal friction peaks for 1.0 mm thickness. It can be seen that the content of solid solution nitrogen atoms in P1 peak is lower than 2 ppm, which is mainly due to the high Al (≥0.035%) and low N (≤60 ppm) in the steel (Table 1) and precipitation in the form of AlN (Figure 6), resulting in basically no free nitrogen atoms in the steel. The solid solution carbon atom and activation energy of P2 peak are lower than those of P3. The activation energy of P3 peak is in the range of 0.84 ± 0.03 eV [29] and 0.76–0.89 eV [25], so therefore, it is certain that the P3 peak is the carbon snoek peak of α-Fe and the P2 peak is the carbon Snoek peak of body centered cubic lattice in which part of Fe is replaced by Si and Mn.

### 3.5. Texture Analysis by XRD

**Figure 9:**
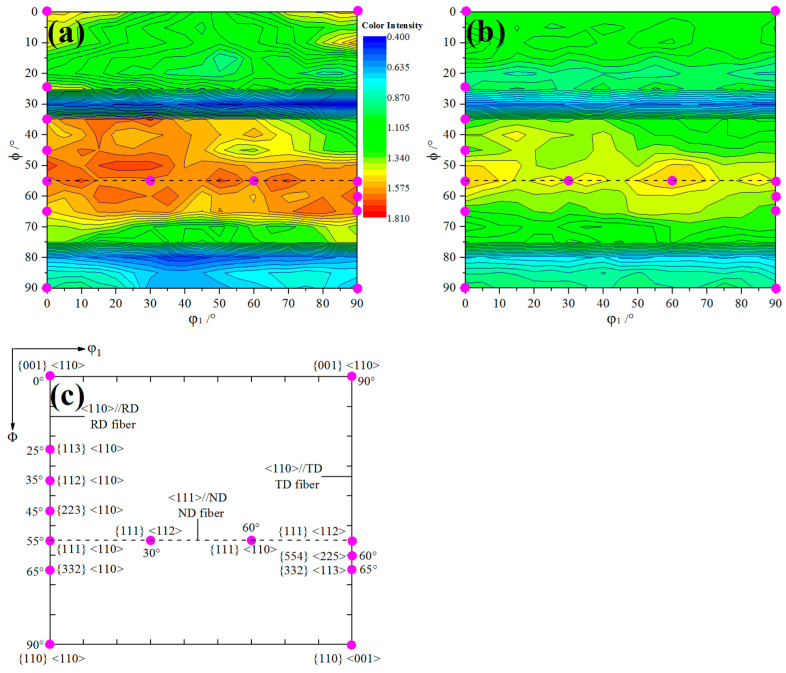
ODF sections (ϕ2 = 45°) of different C content: (**a**) ELC, (**b**) LC, (**c**) the important orientation positions.

Orientation distribution function (ODF) section is the most representative section to reflect a series of important orientation positions. Figure 9 shows the φ2 = 45° sections of the ODF derived from XRD for 1.0 mm thickness strip with different carbon content. It can be seen that although the overall orientations are the same in the two ODF figures, which were mainly composed of incomplete α fiber texture parallel to the rolling direction (RD), incomplete γ fiber texture along the normal direction (ND) and partial fiber texture belonging to the transverse direction (TD). However, there are significant differences in the relative intensity of several important orientation positions such as {001} <110>, {113} <110> and {554} <225>.

**Figure 10:**
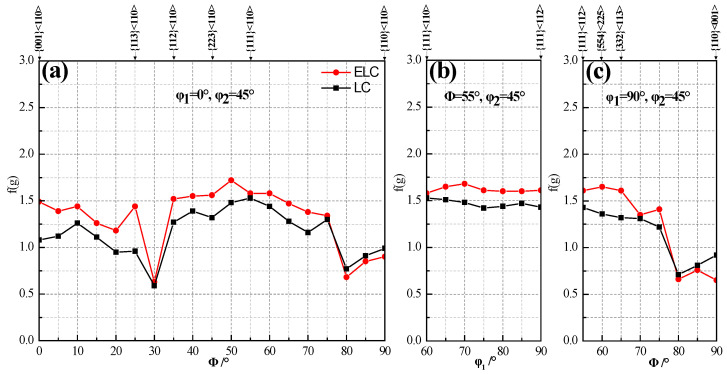
Texture distribution on the different oriented lines: (**a**) RD, (**b**) ND and (**c**) TD.

Figure 10 clearly illustrates the intensity distribution of textures on RD, ND and TD oriented lines. It can be seen that their texture distribution curves are similar and the intensity of ELC is obviously higher than that of LC except for {110} <110> and {110} <001>. On the RD oriented lines, ELC has five orientation positions with about 1.5 intensity, these indicate that ELC has a higher r value than LC, which is consistent with the results in Table 2. The intensity function value on the ND orientation line changes little, indicating that it is difficult to produce ear on the rolling direction of 0°/60°. However, the ELC intensity function values at {554} <225> and {332} <113> on the TD orientation line increase and those of LC decrease, indicating that ELC is more likely to produce ear on the rolling direction of 0°/60°, which can balance ear on the rolling direction of 45° and reduce the earing coefficient [30].

### 3.6. Texture Analysis by EBSD

**Figure 11:**
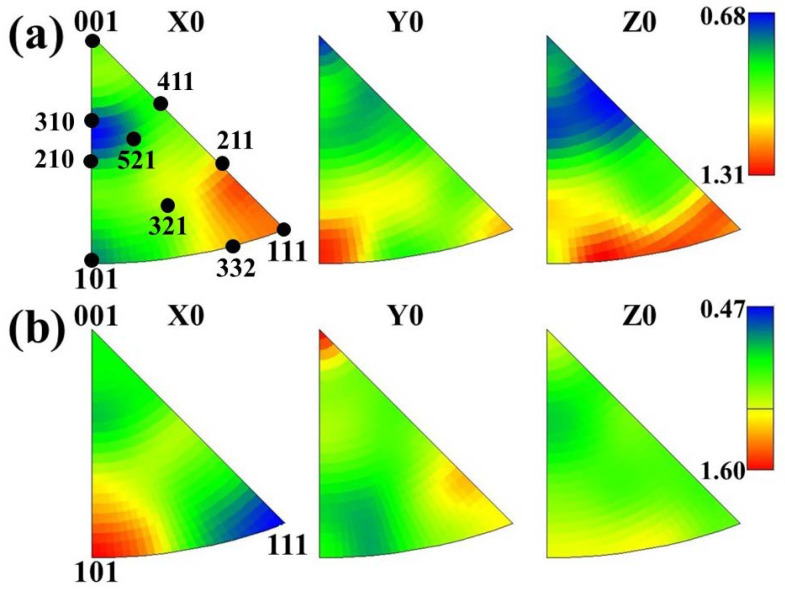
IPF of main texture component with different C content: (**a**) ELC, (**b**) LC.

Although the texture was analyzed macroscopically by XRD, EBSD analysis was carried out along the thickness direction in order to obtain more detailed information about grain orientation. The IPF of main texture component for ELC and LC is shown in Figure 11, It can be seen that the density of {111}, {211} and {001} parallel to the rolling direction (X0) in ELC is higher than that in LC, the density of {111}, {211} and {001} parallel to RD (X0) in ELC is higher than that in LC, the density of {110} parallel to ND (Y0) in ELC is higher than that in LC, and the density of {111} and {332} parallel to TD (Z0) in ELC is higher than that in LC. The change trend is similar to that in Figure 10.

**Figure 12:**
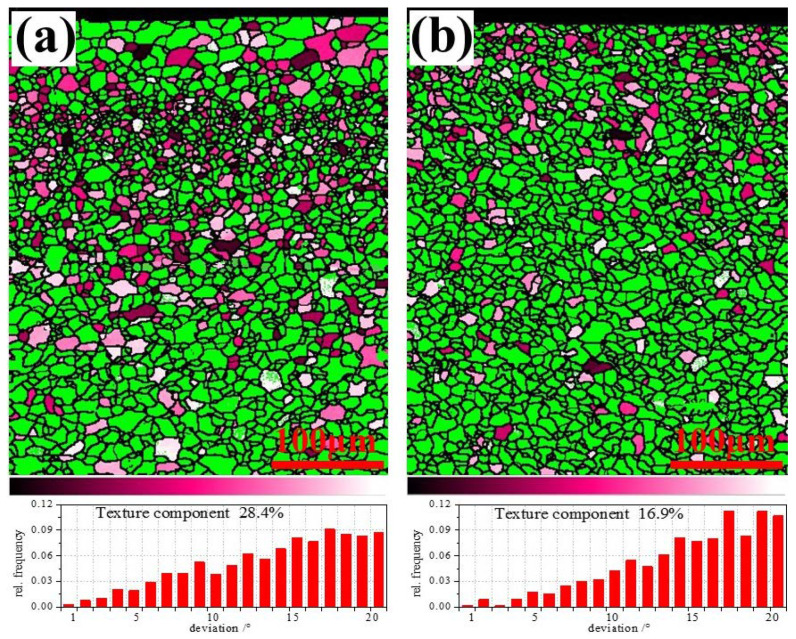
ND fiber texture distribution and fraction of orientation deviation from {111} <uvw> with 20° with different C content: (**a**) ELC, (**b**) LC.

ND fiber texture distribution and fraction of orientation deviation from {111} <uvw> with 20° for ELC and LC is shown in Figure 12. It can be seen that the grain distribution is similar to that in Figure 2 and Figure 3 and the {111} components account for approximately 28.4% for ELC, while this ratio only reaches 16.9% for LC. At the same time, it can also be seen that there are more {111} components account in the fine grain layer of ELC compared with other positions. The stamping formability of IF steel for automobile plate mainly depends on the r value, that is to say, it is better to have more favorable texture components, such as the γ texture ({111}//ND) [31]. Therefore, it can be inferred that the stamping formability of ELC is better than that of LC.

**Figure 13:**
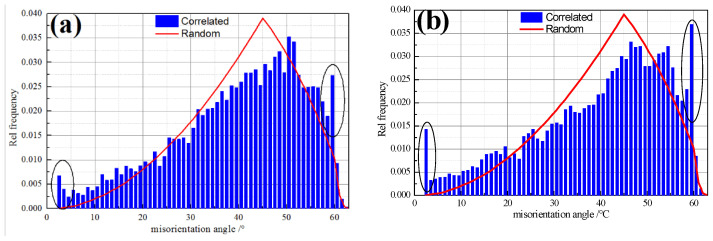
Misorientation distributions of different C content: (**a**) ELC; (**b**) LC.

The misorientation distribution of ultra-thin hot rolled strip for 1.0 mm thickness is shown in Figure 13. Both ELC and LC show the characteristics of random misorientation distribution, but there are significant differences (see black oval in Figure 13). The relative frequency of small angle grain boundary (<10°), especially sub grain boundary (<3°) and 60° of ELC is significantly lower than that of LC, while the relative frequency of 30–40° and about 52° is higher. The increase of small angle grain boundary indicates the increase of dislocation, deformation and strength, the increase of large angle grain boundary indicates high recrystallization. Therefore, it can be seen that there are regions with large deformation degree and regions with high recrystallization degree in ELC and LC, which may be related to the microstructure in Figure 2.

The geometrically necessary dislocations density (ρ^GND^) can be calculated from the local misorientation (θ) using the EBSD orientation data according to the strain gradient theory [32], that is,

ρ^GND^ = 2Δθ/(u b) (4)

where, Δθ is weighted average of θ, b is burgers vector of α-Fe, u is the scanning step, in our experiment, b = 850 μm. The calculated dislocation density of ELC and LC are 8.21 × 10^13^/m^2^ and 9.55 × 10^13^/m^2^ respectively, it is confirmed that the dislocation density of ELC is lower than that of LC. The relevant research showed that the dislocation density of the hot rolled LC steel sheet (0.05% C) by CSP was 2.80 × 10^13^/m^2^ [33].

**Figure 14:**
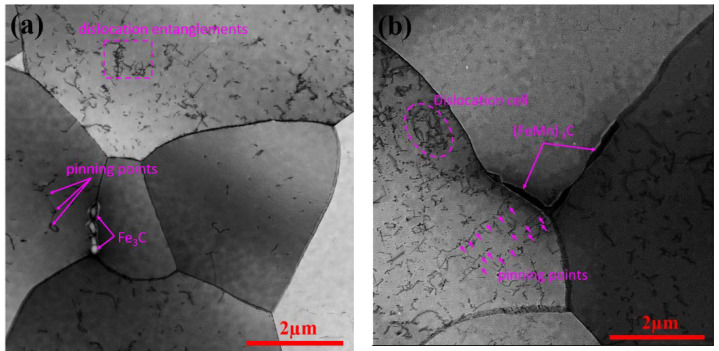
Dislocation structure of different C content: (**a**) ELC; (**b**) LC.

In order to further explain the difference of dislocation density of ultra-thin strips, the dislocation was observed by TEM. Figure 14 shows the dislocation structure for 1.0 mm thickness ultra-thin strip with different carbon content. The dislocation structure is uneven, according to the stress situation, it is considered that there are many dislocations in the pre-eutectoid ferrite grains, while there are few dislocations in the recrystallized ferrite or the eutectoid ferrite. ELC has many pinning points, dislocation lines and a few dislocation entanglements in Figure 14a, but LC has many pinning points, dislocation lines and a few dislocation cells in Figure 14b. It can be clearly seen from Figure 14b that there are a large number of parallel dislocations in the ferrite grains, and the dislocation pinning points are rows of micro precipitates. The dislocation density of LC is higher than that of ELC, which is also one of the reasons why LC has higher upper yield strength than that of ELC. At the same time, a small amount of Fe_3_C (Figure 14a) and (FeMn)_3_C (Figure 14b) are found at the ferrite grain boundaries.

**Figure 15:**
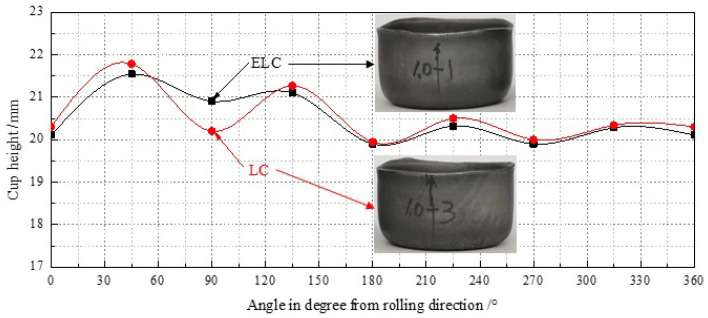
Photograph of draw cups and earing heights at varied angels from rolling direction.

The sheet metal formability is mainly tested by earing test, Hole expanding test and V-shaped bending test. It is considered [34] that the long yield platform is prone to wrinkles on the steel surface in the process of stamping or bending deformation, which affects the forming effect and appearance. Therefore, the formability of ultra-thin strip by ESP was tested. Figure 15 shows the photograph of draw cups and earing heights at varied angels from rolling direction. It is shown that the ears of ELC were smaller than those of LC, there are four ears produced during drawing and their earing direction was 45°, and there are no obvious defects such as wrinkles, localized bands of plastic deformation on the appearance of the draw cups.

Several important indexes to characterize the earing propensity are listed in Table 5 in which $\bar{h}$t, $\bar{h}$v, $\bar{h}$e, Δhmax, and Ze meant the average height of ear peak, ear valley, earing, maximum ear height, and earing coefficient, respectively. Although Ze of both meets the user’s requirements of less than 5%, but, $\bar{h}$t, $\bar{h}$e, Δhmax, and Ze of ELC were lower than those of LC, so ELC has better earing performance than LC.

**Figure 16:**
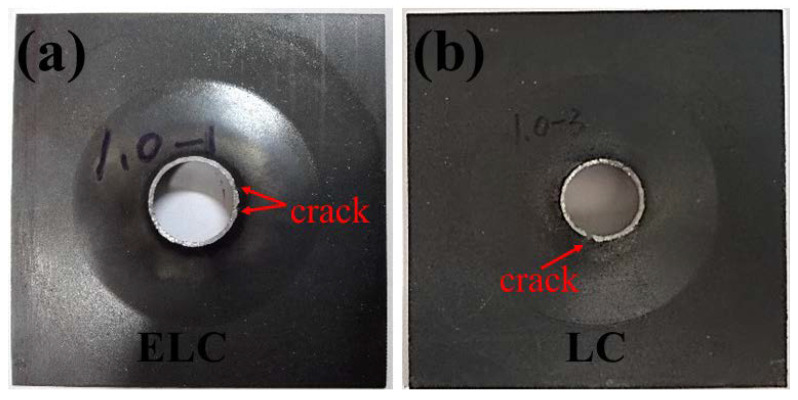
Photograph after hole expanded with different C content: (**a**) ELC; (**b**) LC.

The morphology after hole expanded for ELC and LC is shown in Figure 16. It can be seen that the hole diameter of ELC was larger than that of LC, and two small cracks appeared in ELC, while a large crack appeared in LC, which were a typical β fractures exceeding their elongation. The calculated limiting hole expansion ratio are 79.1% and 61.4% respectively, both meet the delivery requirement of more than 50%.

**Figure 17:**
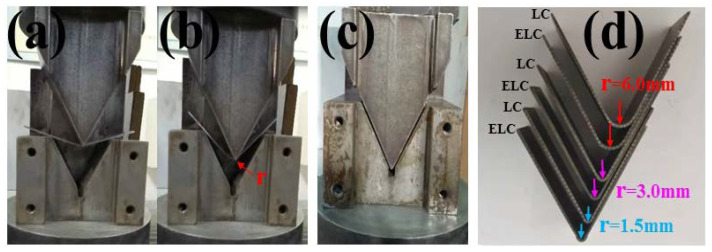
V-shaped bending process and bended parts with different C content: (**a**) 150°, (**b**) 100°, (**c**) 60°, (**d**) bended parts.

Figure 17 shows the V-shaped bending process and bending parts. The downward speed of the upper mold was 1 mm/s, holding for 30 s after reaching the specified position, and then the upper mold rises quickly, remove the sample and measure its angle. The measured angles were all 60° and the samples under different bending radium (1.5 mm, 3.0 mm, 6.0 mm) did not rebound, as shown in Figure 17d, and there were no obvious defects such as wrinkles on the appearance of the bended parts.

From the above experimental results, it can be considered that the 1.0 mm ultra-thin hot rolled steel strip by ESP has good earing behavior, hole expansion ratio and bending performance, can meet the requirements of tropical cooling, is conducive to energy conservation and emission reduction, and achieves carbon neutralization as soon as possible.

## 4. Discussion

There are significant differences in the microstructure and mechanical properties of the hot-rolled strip by conventional hot rolling, thin slab casting and rolling and ESP due to their different hot rolled process [35,36]. Due to the uniformly distributed microstructure observed in the 1.0 mm hot rolled strip by compact strip production [3], so it is considered that this phenomenon is related to the unique ESP process, especially induction heating before finishing rolling. The penetration depth (δ) is calculated [37] by:

(5) $$I_{x}=I_{0}\;e^{-x/\delta },$$

where x is the depth from the surface; I_x_ is the current density at the depth of x in the intermediate slab; I_0_ is the current density on the intermediate slab surface.

(6) $$Q_{x}=k\;p_{x}\;t=k\;I_{x}^{2}\;R\;t,$$

where Q_x_ and P_x_ are heat and power at the depth of x in the intermediate slab, k is efficiency, t is the heating time, R is resistance of slab. The heat conduction equation considering only the thickness direction is as follows:

(7) $$\frac{\partial T}{\partial t}=\alpha \frac{\partial^{2}T}{\partial y^{2}}+\frac{q}{\rho \;c},$$

where T is the temperature of rolling piece, t is the time, y is the coordinate in the thickness direction, q is calorific value per unit area and time, ρ is density and c is specific heat of rolled piece. The effect of inductive heating on the microstructure evolution of ultra-thin hot rolled strip was mainly shown in the following aspects:

- Because of the skin effect and penetration depth, the temperature gradient of the intermediate slab from the upper surface to the thickness center after induction heating shows a parabolic exponential relationship, which is similar to the square of induced current density. As the slab leaves the induction heating device and contacts with the air, the intermediate slab temperature decreases according to the above heat conduction. Under the combined action of these two temperature gradient distributions, the temperature distribution from surface to the thickness center is parabolic, so a high temperature area appears in the near surface of intermediate slab.
- In the subsequent finishing rolling process, due to its high temperature and low deformation resistance, the microstructure in the near surface area is more prone to dynamic recrystallization and grain refinement, that is, the fine grain layer appears in the near surface area, the microstructure of the inner area is transformed such as traditional rolling to obtain a medium grain area in the center. For ELC, because the precipitates are small and cannot prevent the growth of surface grains, the surface coarse grain area is formed. While for LC, the precipitates are coarse and the transformed pearlite prevents the growth, resulting in the similar fine grain microstructure as that of the near surface. A similar non-uniform microstructure was observed on the steel plate with a thickness of 1.5 mm by ESP, and it was considered that the non-uniformity of temperature field distribution of intermediate slab had a corresponding relationship with the non-uniformity of microstructure in the thickness direction of strip [38].

The reasons why ultra-thin hot rolled wide strip steel has high upper yield strength and long percentage yield point extension are as follows:

- In the elastic deformation stage, the stress and strain increase proportionally. At the beginning of plastic deformation, the density of movable dislocations is small, with the increase of stress, more dislocations appear, causing the dislocation density and the interaction between dislocations increases rapidly. At the same time, by diffusing to the dislocation sites, solute atoms play the role of pinning or segregation to hinder the movement of dislocations [15]. These all cause the stress to increase and reach a peak quickly, that is, the high upper yield strength appears. At the same time, because the grain boundary is an obstacle to dislocation movement, the finer the grain is, the more difficult the dislocation moves, and fine grain strengthening can improve the strength and plasticity [39], therefore, the strength and plasticity of LC are higher than those of ELC.
- When the dislocations break away from the dislocation locking, the dislocation movement is easier and the stress drops, and the lower yield point appears. The repeated locking and unlocking process before getting rid of the obstruction leads to discontinuous yield, that is, the emergence of yield platform. It is generally believed [40] that the more interstitial atoms in the Cottrell atmosphere, the more dislocations are locked, the greater the resistance of dislocation movement and the longer the percentage yield point extension. It is also considered [41] that the case of ferrite-cementite steels with very small cementite particles, continuous yielding cannot be observed. The latest research [42] shows that when the applied stress increases enough to activate a source such as the incoherent α/θ interface or grain boundary, a large amount of dislocation can be emitted by the high applied stress, leading to the yield drop; after getting a dislocation density high enough to have plenty of plastic strain, and then yielding occurs in Grain 2, resulting in discontinuous yielding. Because there are more solid solution carbon atoms, dislocations and small precipitates in ESP ultra-thin strip than those in traditional hot rolled strip, a higher percentage yield point extension is obtained.

The most important factor that affects the formability is the distribution of texture in steel strip, different textures have different effects on formability. In order to better evaluate the earing behaviors, the earing extent index (Q) is used to quantitatively describe the relative earing tendency of each texture component. Table 5 shows $\bar{r}$, Δr, Q and earing direction for main texture components [30,43]. According to Table 6, texture components such as {111} <110>, {111} <112> and {554} <225> contribute great to $\bar{r}$ and little or minor to Δr. At the same time, texture balances between {hkl} <110> and {554} <225> or {332} <113> was quite effective in eliminating earing. On the whole, the influence of ELC texture on $\bar{r}$ and Δr is better than those on LC, so the formability of ELC is better than that of LC.

The reasons why the ELC had good formability were that it had:

- The important indexes to measure formability are r and Δr. In order to obtain steel strip with good formability, we want it to have as high $\bar{r}$ and Δr close to 0 as possible. The r0, r45 and r90 and $\bar{r}$ of ELC are about 0.1 higher than those of LC, and Δr is about 0.1 lower than that of LC. Therefore, from a macro point of view, the formability of ELC is better than that of LC.
- In terms of texture control, high r and low Δr are obtained by promoting {111} texture and restrict {001} texture [44]. During the forming process, the {hkl} <112> grains parallel to the rolling direction will continuously turn to {hkl} <110> along the slip plane until all {hkl} <112> grains turn to {hkl} <110>, then the limit of grain deformation has been reached. At the same time, the content of {111} <uvw> gradually decreases resulting in the gradual decrease of favorable γ texture. The content of {hkl} <110> and {111} <112> of ELC was higher than those of LC, so the formability of ELC is better than that of LC.

## 5. Conclusions

The 1.0 mm thickness ultra-thin hot rolled strip produced by endless roll technology was studied, and a detailed investigation of the microstructural characterization. Characteristics of cementite and precipitates, mechanical properties, internal friction peaks, texture characterization by XRD and EBSD, and formability including earing behavior, hole expanding ratio and V-shaped bending properties was carried out. The following conclusions were drawn:

- The microstructure of ultra-thin strip along the thickness direction is composed of multi-layer areas with different grain sizes and thicknesses, which is related to the uneven temperature distribution in the thickness direction caused by induction heating process of ESP. The high-temperature area formed at the near surface area occurs austenite recrystallization and grain refinement in the finishing rolling process. Subsequently, the microstructure at the surface of ELC and LC is different due to the influence of pearlite transformation and precipitate size.
- The stress–strain curve of ultra-thin hot rolled strip shows a typical discontinuous yield phenomenon, which has an upper yield strength higher than the tensile strength and a percentage yield point extension of about 10%, which may be directly related to the high content of solid solution carbon atoms, high dislocation density and the locking effect of carbon atoms and precipitates on dislocations. The strength and elongation of LC are higher than that of ELC, which is mainly affected by fine grain strengthening and dislocation strengthening, but LC has higher upper yield strength and percentage yield point extension, smaller r value and larger Δr value, so the formability of LC is worse than that of ELC.
- The ultra-thin hot rolled strip has good earing behavior, limiting hole expansion ratio and bending performance, which can meet the requirements of replacement cold rolled strip by hot rolled strip. The formability of ultra-thin strip ELC is better than that of LC mainly related to the content of {hkl} <110> and {111} <112> of ELC was higher than those of LC.

## Figures and Tables

**Table 1:** Chemical composition of investigated materials (mass fraction, %).

Steel	C	Si	Mn	P	S	Al	N	Fe
ELC	0.012	0.031	0.124	0.018	0.002	0.037	0.0053	balance
LC	0.044	0.032	0.120	0.015	0.001	0.038	0.0055	balance

**Table 2:** Process parameters of the ESP line for 1.0 mm thickness.

Steel	Casting Speed	RET	RDT	IET	IDT	FDT	CT
	(m·min^−1^)	(°C)					
ELC	5.50	991	984	920	1110	845	665
LC	5.49	987	981	916	1113	846	664

**Table 3:** r value and n value of different C content.

Steel	r_0_	r_45_	r_90_	$\bar{r}$	Δr	n_0_	n_90_
ELC	0.875	1.069	1.028	1.010	−0.235	0.229	0.217
LC	0.749	0.993	0.926	0.915	−0.310	0.228	0.215

**Table 4:** Important indexes characterizing the internal friction peaks of different C content.

Steel	Peak	Tm	fm	$Q_{max}^{-1}$	[C] or [N]	H	
		/K	/Hz	/10^−4^	/ppm	/KJ/mol	/eV
ELC	P1	287.36	2.80	0.55	[N]:0.73	67.84	0.71
	P2	310.76	2.80	4.26	[C]:5.66	73.57	0.77
	P3	341.26	2.79	14.30	[C]:19.02	81.06	0.84
LC	P1	293.86	2.65	1.26	[N]:1.68	69.56	0.72
	P2	314.06	2.64	2.59	[C]:3.45	74.52	0.78
	P3	344.86	2.64	7.69	[C]:10.23	82.11	0.85

**Table 5:** Several important indexes to characterize the earing propensity.

Steel	$\bar{h}$t (mm)	$\bar{h}$v (mm)	$\bar{h}$e (mm)	Δhmax (mm)	Ze (%)	Earing Direction (°)
ELC	20.81	20.20	0.61	1.64	3.02	45
LC	20.97	20.11	0.86	1.78	4.29	45

**Table 6:** The $\bar{r}$, Δr, Q and earing direction for main texture components.

Texture Component	$\bar{r}$	Δr	Q	Earing Direction (°)
{001} <110>	0.4	−0.8	0.782	45
{114} <110>	1.2	−2.1	not available	45
{113} <110>	1.0	−1.7	not available	45
{112} <110>	2.07	−2.69	not available	45
{223} <110>	2.5	−2.0	not available	45
{111} <110>	2.62	0.01	0.071	No ears
{111} <112>	2.62	0.01	0.071	No ears
{554} <225>	2.61	1.09	0.207	0/90
{332} <113>	2.7	1.9	not available	0/60
{110} <001>	5.04	8.95	0.783	0/90
{001}<100>	0.41	0.75	0.761	0/90

## Data Availability

The data presented in this study are available on request.
